# Supplementary material for: Reconciling metal–silicate partitioning and late accretion in the Earth
Source: Nat Commun. 2021 May 18;12:2913. doi: 10.1038/s41467-021-23137-5 (PMC8131616; doi:10.1038/s41467-021-23137-5)
Supplement: Supplementary file 1 — Supplementary Information [file 41467_2021_23137_MOESM1_ESM.pdf]

SUPPLEMENTARY INFORMATION

Supplementary Notes 1: Infinite Dilution Correction

In order to apply these partitioning results to the low platinum concentrations relevant to core formation, we applied an activity correction to account for non-ideal mixing behavior of high platinum content in the alloy. The activity coefficients of the components in a solution depend on concentration and are related by the excess Gibbs free energy of mixing of the solution. The activity coefficients of Pt in the alloy were determined using a Margules-type formulation (e.g., *1*) for a binary FePt mixture.

The excess free energy of mixing, G_M^{xs} at given pressures (P) and temperatures (T) for a solution can be expressed as follows (*1, 2*):

$$G_M^{xs} = \Delta H_M - T\,\Delta S_M^{xs} + (P-1)\Delta V_M^{xs} + (P-1)\,\Delta V \qquad \text{(Eq. S9)}$$

Where ΔH_M is the enthalpy of mixing, ΔS_M^{xs}, the excess entropy of mixing and ΔV_M^{xs} is the excess volume of mixing. The G_M^{xs} of a binary mixture, can be also be expressed in terms of the component molar fractions (X) and Margules mixing constants (W) for the components, e.g.,

$$G_M^{xs} = X_{Fe}X_{Pt}\,W_{FePt} \qquad \text{(Eq. S10)}$$

The Margules mixing parameter can be re-expressed as:

$$W_{FePt} = W^{H,1\,bar} - TW^S + (P-1)W^V \qquad \text{(Eq. S11)}$$

Where $W^{H,1\,bar}$, W^S and $W^V$are related to the excess enthalpy of mixing, excess entropy of mixing, and excess volume of mixing. Equation (S11) is analogous to the G_M^{xs}, allowing the activity coefficients, γ_{Pt}^{∞} to be determined from the following relationship (*1*):

$$RT\ln(\gamma_{Pt}) = W_{PtFe}X_{Fe} - G^{xs} \quad \text{(Eq. S12)}$$

The interaction parameters for liquid binary Fe–Pt mixtures used to correct the measurements in the current study and the literature data (*2, 3*) are from table EA.3 of (*2*) and (*4*) (W^H (FePt) = -268287, W^S (FePt) = -72, W^V (FePt) = 0.08, W^H (PtFe) = -206287, W^S (PtFe) = -72, W^V (PtFe) = 0.08, Tref = 2050 K). Two of the previous studies on Pt partitioning used starting alloys that were iridium-rich and iron–poor and required large activity corrections relative to those of the present study. The interaction parameters for ternary mixtures Fe–Pt–Ir used to correct these datasets (*5, 6*) were obtained from the values compiled in (*2, 7*).

The activity coefficients obtained were then used to extrapolate the measured Pt partition coefficients to values corresponding to infinite dilution, hence removing the effect of non-ideal mixing (See Supplementary Table 1). Supplementary Figure 5 shows that the Pt activities obtained for the alloys have a roughly linear relationship with the Pt concentration in the alloy.

Supplementary Figures

Supplementary Figure 1: Laser heating temperature ramp for run # 4

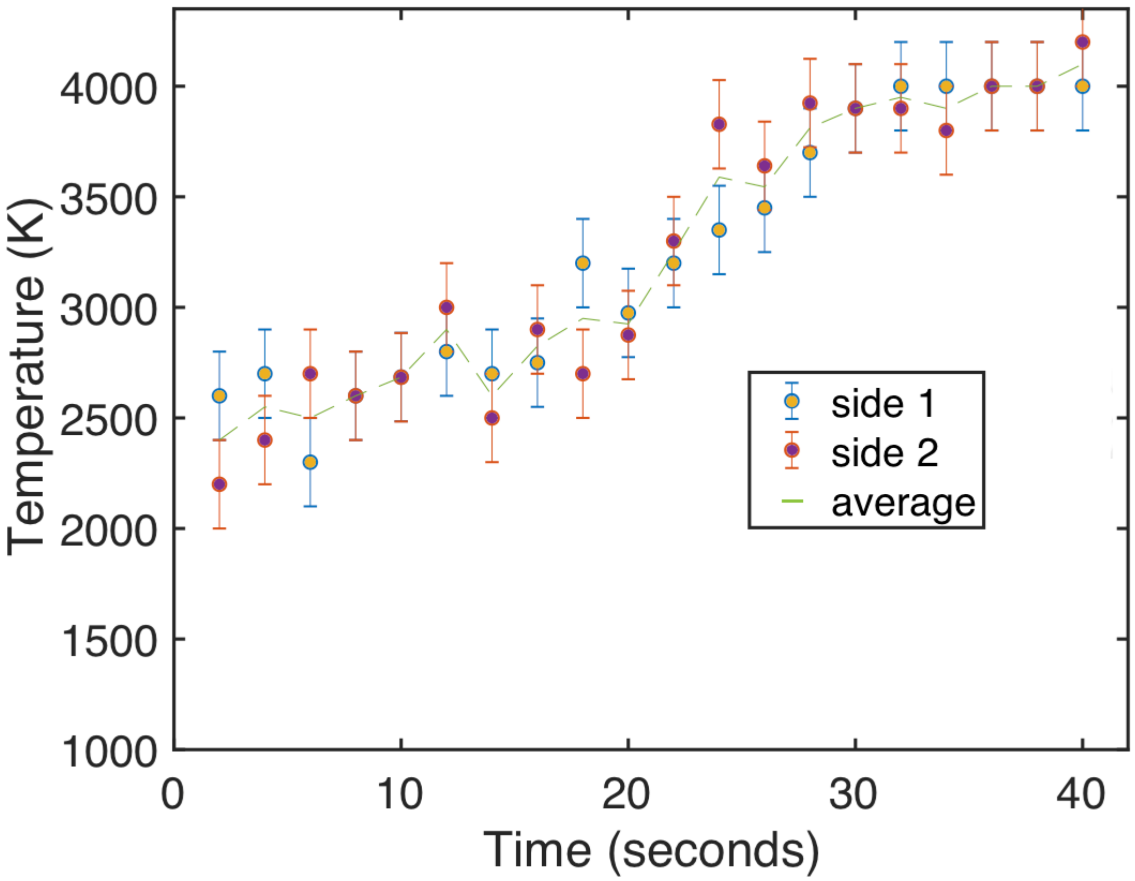

The temperatures recorded from the two sides of run # 4 during heating over the last 40 seconds of the experiment. Temperatures were recorded approximately every 2 seconds. The error bars incorporate an analytic uncertainty and the standard deviation in highest temperatures from several experiments. The final average quench temperature for this experiment is 4100 ± 200 K.

Supplementary Figure 2: Geometry of FIB lamella for NanoSIMS analysis

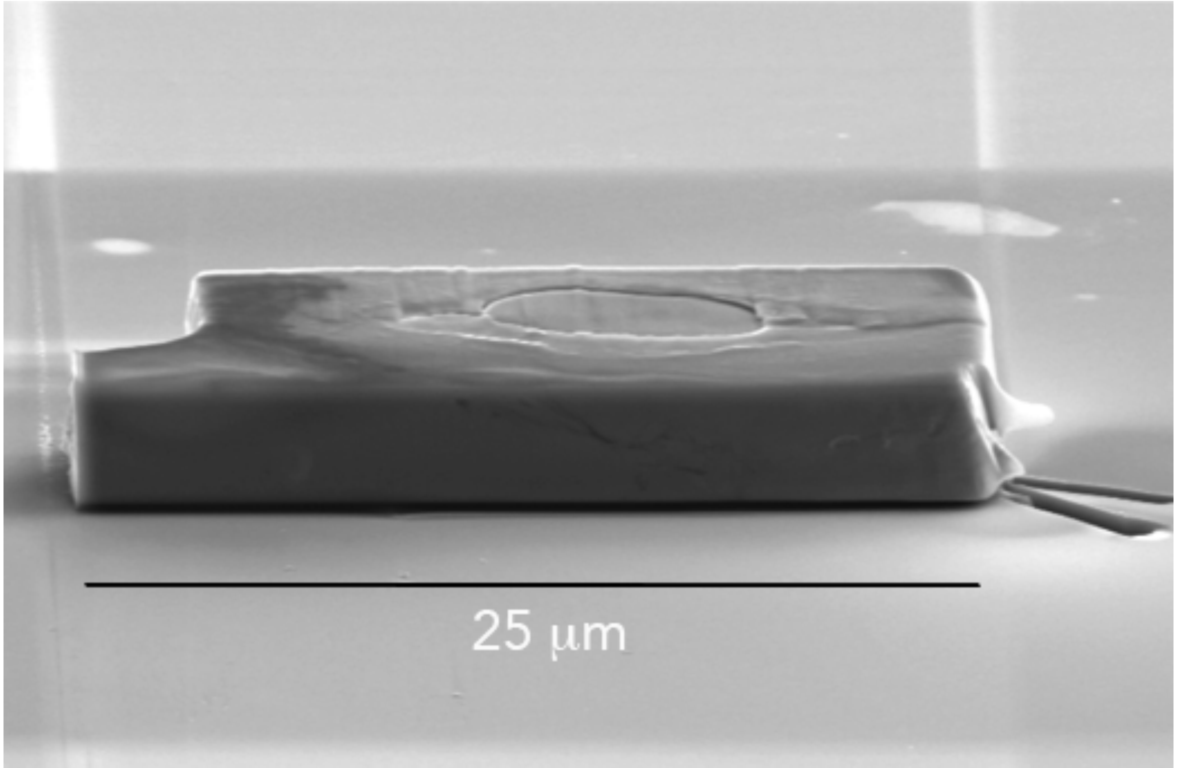

Electron image of run # 5 after FIB preparation. The lamella is deposited flat onto a silicon wafer, in a geometry that is optimal for NanoSIMS analysis (i.e., prevents edge effects due to voids when the section is attached to a copper classical TEM grid). Samples were attached by an electron curing glue from Kleindiek Nanotechnik.

Supplementary Figure 3: Calibration for NanoSIMS measurement of platinum

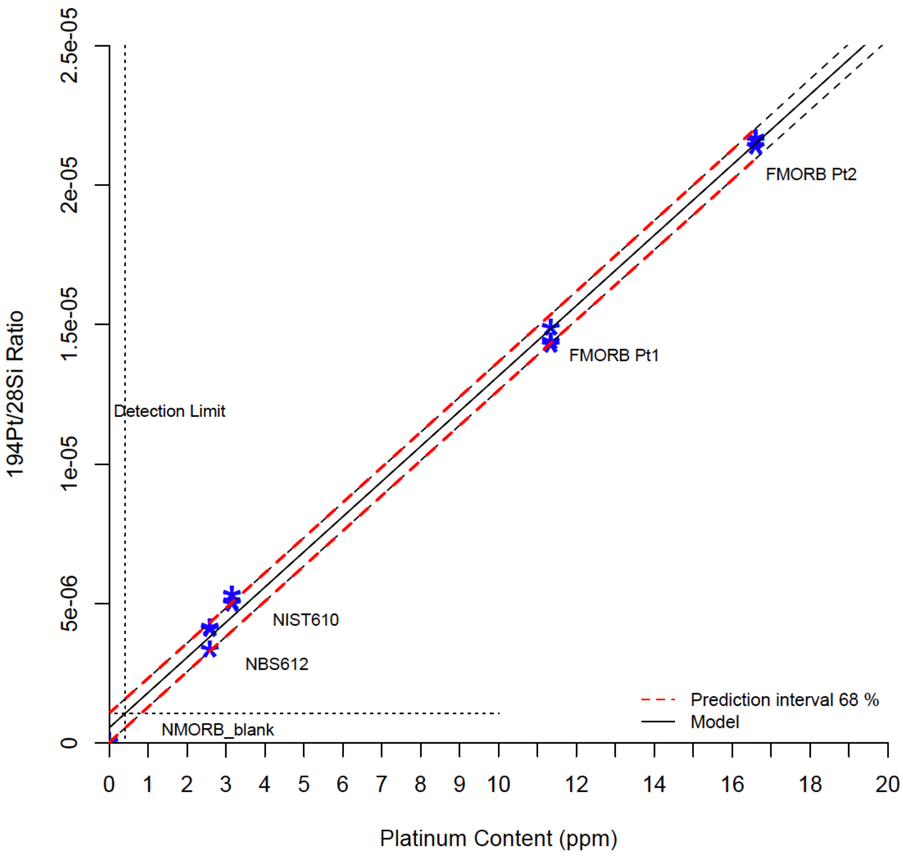

The concentrations of platinum in silicate standards measured by LA-ICPMS (x-axis) and $^{194}Pt/^{28}Si$ counts measured by NanoSIMS (y-axis). The solid black line was obtained from a regression analysis of the standard measurements. An inverse regression model was then used determine the platinum compositions from $^{194}Pt/^{28}Si$ counts of the quantitative NanoSIMS maps. The dashed red lines are the prediction intervals, and the black dotted lines are detection limits determined from the inverse modeling.

Supplementary Figure 4: Partial equilibration core formation model result

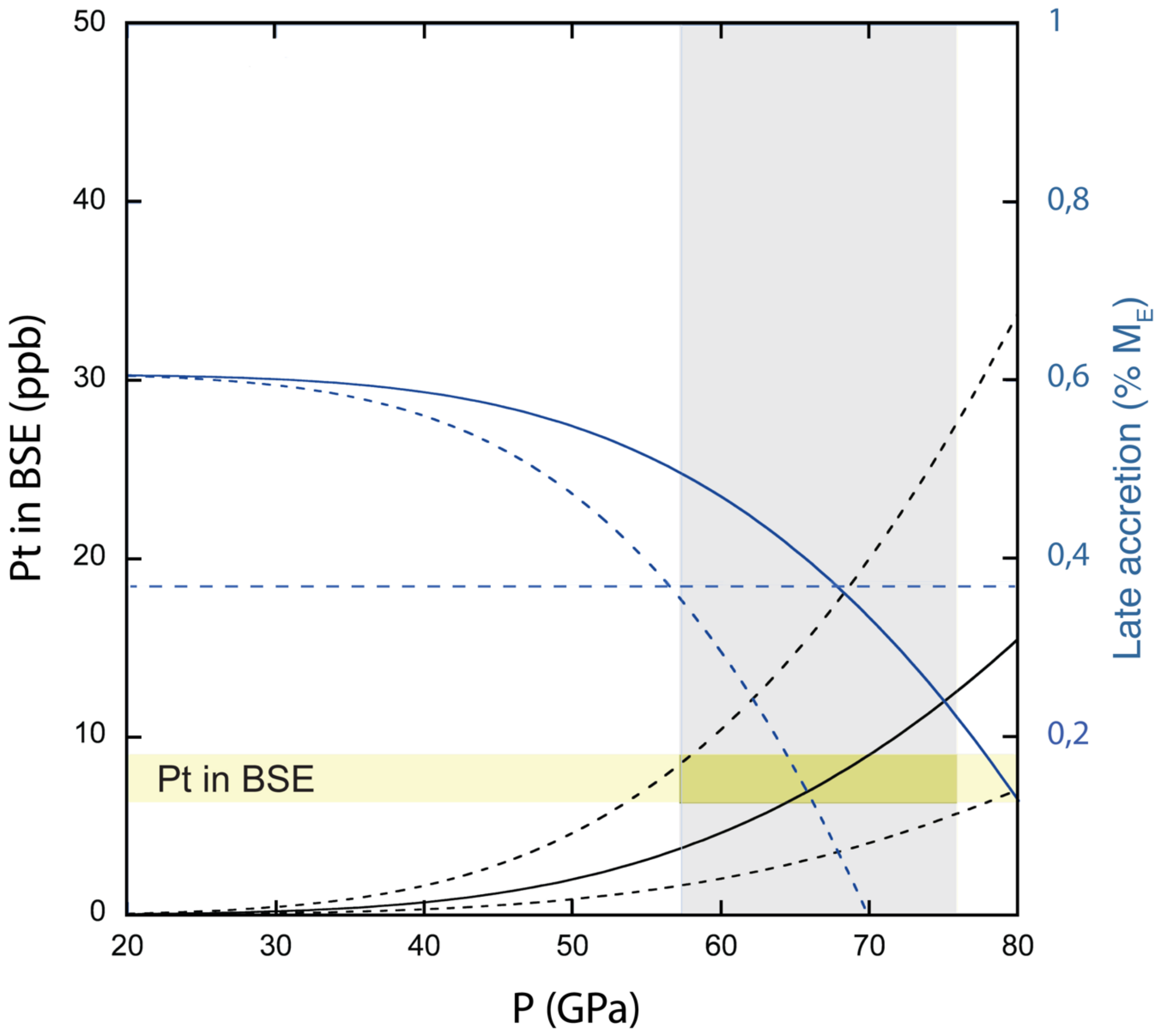

Platinum accumulated in the mantle versus final equilibration pressure. Platinum (black curve) is lowered by reduced core-mantle equilibration efficiency relative to full equilibration (See Figure 3a). Between 65 and 72 GPa, this result is compatible with the observations of platinum in the mantle. Above ~ 68 GPa, there is too much platinum relative to observations and not enough late accretion mass (blue curve) to account for the other highly siderophile elements (blue horizontal line). The solid blue line is the late accretion mass estimates and dashed blue line is the lower

error envelope (derived from uncertainty in the regression model). This scenario is also not compatible with the mantle's moderately siderophile abundances.

Supplementary Figure 5: Platinum activity versus concentration in the metal

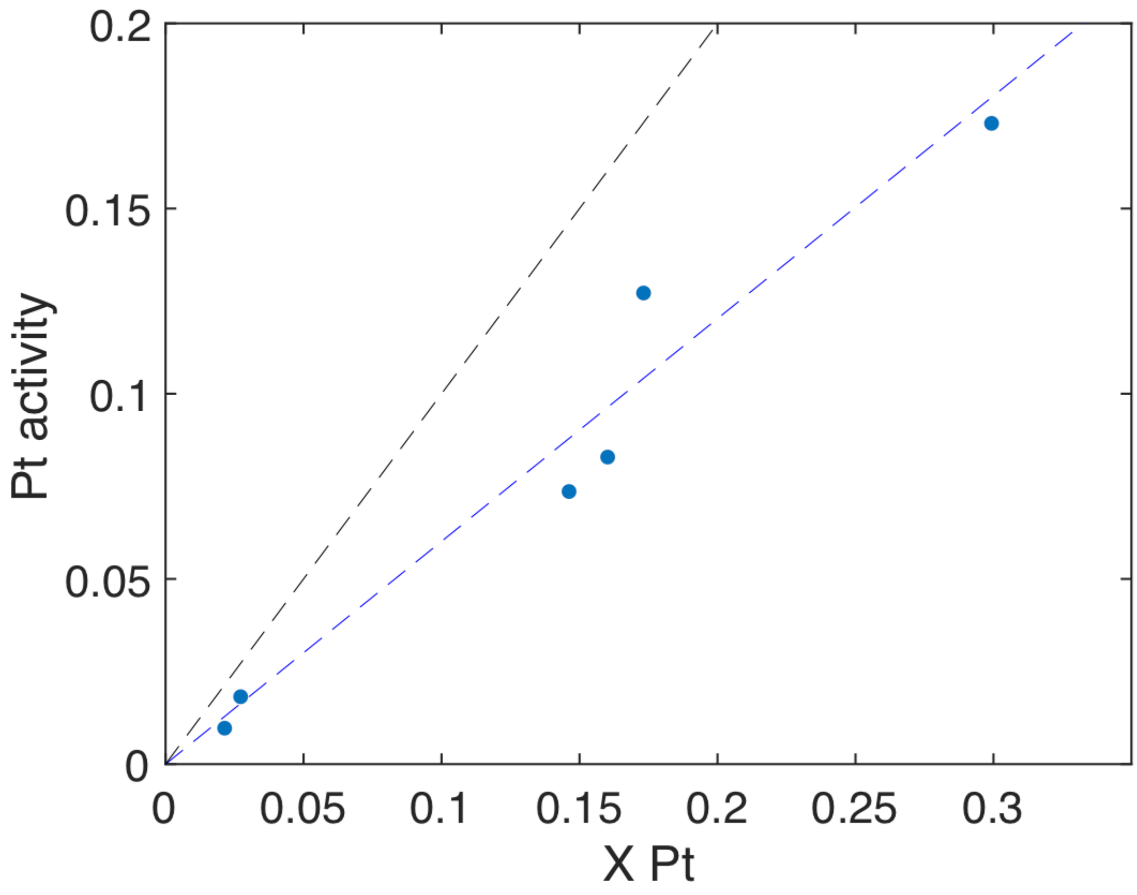

The blue dots show the calculated platinum activity against measured concentrations in mole fractions of the main metallic component of samples in this study. The blue dashed line is a linear fit to the data and black dashed line shows ideal mixing behavior.

Supplementary Tables

Supplementary Table 1: Experimental Conditions

Run	Pressure (GPa) (± 5)	Temperature (K) (± 200)	D (Pt) (measured)	D^0 (Pt) (infinite dilution corrected)	Δ IW	X $FeO_{silicate}$	X Fe_{metal}	X Si_{metal}	X O_{metal}	X S_{metal}	X Pt_{metal}
1	43	4000	163.73	160 ±19	-0.99	0.2336 ±0.004	0.7342 ±0.007	0.0458 ±0.002	0.2023 ±0.010	0.0149 ±0.001	0.1462 ±0.001
2	54	3600	423.01	414 ±113	-1.11	0.1705 ±0.006	0.6102 ±0.002	0.0298 ±0.001	0.2225 ±0.007	0.1088 ±0.0002	0.1603 ±0.002
3	65	3900	449.37	385 ±203	-0.96	0.1778 ±0.006	0.5350 ±0.017	0.0315 ±0.004	0.3082 ±0.045	0.0939 ±0.004	0.2993 ±0.003
4	75	4100	152.74	135 ± 33	-0.67	0.1918 ±0.012	0.4169 ±0.016	0.0255 ±0.002	0.2106 ±0.028	0.1524 ±0.004	0.1732 ±0.005
5	85	3900	49.56	54 ± 9	-0.78	0.2491 ±0.011	0.6116 ±0.011	0.0144 ±0.001	0.2047 ±0.037	0.1251 ±0.002	0.0272 ±0.0006
6	111	4300	21.22	16 ± 2	-1.26	0.1613 ±0.018	0.6857 ±0.009	0.0667 ±0.003	0.1584 ±0.010	0.0289 ±0.001	0.0445 ±0.0006

Summary of the experimental conditions (pressures, temperatures, oxygen fugacities (interms of Δ IW), the measured and infinite dilution corrected platinum metal-silicate partition coefficients, D (Pt) and D^0 (Pt), for each of the six runs in this study. The FeO content of the silicate, the Fe and the light element composition of the main metal reservoir are also given in molar fractions (X) for each run.

Supplementary Table 2: Starting silicate composition

**Oxides**	SiO_2	FeO	MgO	Al_2O_3	CaO	Na_2O	TiO_2	MnO	Total
**wt. %**	49.74 ± 0.49	9.73 ± 0.28	8.46 ± 0.12	15.89 ± 0.23	11.74 ± 0.19	2.69 ± 0.09	1.33 ± 0.13	0.18 ± 0.06	100.1

The major oxide composition in weight percent of the natural MORB starting material used in this study. The sample was quantified by electron microprobe analysis and minor elements (not shown) were included in the total.

Supplementary Table 3: Standard platinum concentrations

**Standards**	**Platinum (ppm)**
NMORB-blank1	0.005 ± 0.007
NIST 612	2.59 ± 0.03
NIST 610	3.15 ± 0.08
FMORB-Pt1	11.23 ± 0.12
FMORB-Pt2	16.38 ± 0.16

The platinum concentrations (in ppm) of silicate glass standards as measured by LA-ICPMS. NIST 610 and 612 are certified NIST standards (*8*).

Supplementary Table 4: Compositions of the experimental run products.

Runs	1	2	3	4	5	6
**Pressure (GPa)**	**43** (± 5)	**54** (± 5)	**65** (± 5)	**75** (± 5)	**85** (± 5)	**111** (± 5)
**Temperature (K)**	**4000** (± 200)	**3600** (± 200)	**3900** (± 200)	**4100** (± 200)	**3900** (± 200)	**4300** (± 200)
Silicate Composition						
MgO	5.85± 0.23	7.09 ±0.44	6.39 ±0.49	5.31 ±0.33	3.93 ±0.23	6.75 ±0.16
SiO_2	37.14 ±0.24	37.95 ±0.3	36.72 ±0.28	36.05 ±0.77	27.66 ±0.83	37.06 ±1.80
Al_2O_3	17.26 ±0.18	19.92 ±0.5	22.02 ±0.58	22.30 ±0.58	23.07 ±0.87	24.31 ±0.69
CaO	2.97 ±0.08	4.27 ±0.19	3.26 ±0.13	3.01 ±0.10	2.52 ±0.08	2.81 ±0.12
Na_2O	3.60 ±0.21	2.98 ±0.07	4.33 ±0.09	3.70 ±0.39	3.67 ±0.28	2.57 ±0.38
K_2O	1.33 ±0.10	1.25 ±0.08	0.91 ±0.11	1.11 ±0.25	1.57 ±0.42	0.75 ±0.06
FeO	29.17 ±0.50	21.53 ±0.80	22.77 ±0.76	24.30 ±1.55	30.84 ±1.32	20.37 ±2.28
MnO		0.44 ±0.07	0.40 ±0.06	0.30 ±0.06	0.59 ±0.06	0.30 ±0.07
TiO_2	2.50 ±0.13	3.94 ±0.05	2.76 ±0.04	3.38 ±0.08	5.74 ±0.24	4.57 ±0.28
PtO_2	0.17 ±0.11		0.27 ±0.18	0.21 ±0.21	0.21 ±0.08	0.50 ±0.28
S	0.01 ±0.02	0.14 ±0.01	0.16 ±0.01	0.34 ±0.20	0.19 ±0.05	
Pt (NanoSIMS)	0.3066 ±0.0356	0.1299 ±0.0354	0.2316 ±0.1221	0.3902 ±0.0954	0.1457 ±0. 0638	0.7190 ±0.0719
Total	100	99.51	100	100	100	100
Metal Composition						
O	4.34 ±0.21	4.8 ±0.15	5.03 ±0.74	5.04 ±0.67	6.86 ±1.25	4.74 ±0.29
Fe	54.96 ±0.50	45.94 ±0.14	30.45 ±0.96	34.85 ±1.33	71.56 ±1.30	71.68 ±0.89
Si	1.73 ±0.09	1.13 ±0.05	0.90 ±0.11	1.07 ±0.09	0.85 ±0.05	3.51 ±0.18
Mn			0.28 ±0.11	0.22 ±0.09	0.41 ±0.26	0.72 ±0.01
Al		0.72 ±0.10	0.36 ±0.04	0.47 ±0.08	0.36 ±0.05	0.31 ±0.04
Mg		0.29 ±0.05	0.33 ±0.05	0.24 ±0.06	0.37 ±0.03	0.13 ±0.04
S	0.64 ±0.04	4.7 ±0.01	3.07 ±0.15	7.31 ±0.19	8.40 ±0.16	1.73 ±0.04
**Pt**	38.24 ±0.31	42.15 ±0.5	59.52 ±0.66	50.60 ±1.43	11.10 ±0.27	16.24 ±0.25
**Total**	99.90	99.73	100	100	100	100

The compositions of the quench silicate and quench metal in weight percent for each of the six runs in this study. Major element compositions were obtained by EDX and the platinum concentrations in the silicate were obtained by NanoSIMS. Minor elements, not shown were included in the total.

Supplementary Table 5: NanoSIMS platinum measurements for silicate part of run # 5

ROI	$^{194}Pt/^{28}Si$	Pt concentration (ppm)
1	0.00167354	1326
2	0.00181605	1439
3	0.00064475	511
4	0.00120162	952
5	0.00181349	1437
6	0.00295326	2341
7	0.00159775	1266
8	0.00301076	2386
**Average**		1457
**1 σ**		638

Pt/Si ratios and derived platinum concentrations from several regions in the quench silicate of run # 5. Their average is used as the concentration of platinum in the quench silicate part of the sample and their standard deviation is the uncertainty. This procedure was repeated to determine the concentration of platinum in the quench silicate portions of all the samples.

Supplementary Table 6: Compositions of nanoparticles in weight %

Nanoparticles	Fe	O	Si	Pt	Fe/Pt
**1**	77.97	9.72	5.43	6.89	11.32
**2**	79.91	8.27	6.33	5.49	14.56
**3**	80.57	6.99	4.09	8.35	9.65
**4**	77.6	11.05	5.92	5.42	14.32
**Average**	79.01	9.01	5.44	6.54	12.46

Compositions of nano metallic particles obtained with energy dispersive X-rays (EDX) in a transmission electron microscope (TEM) from the quench silicate section of run # 1 (See main text Figure. 1e).

REFERENCES

1. B. Mukhopadhyay, S. Basu, M. J. Holdaway, A discussion of Margules-type formulations for multicomponent solutions with a generalized approach. *Geochimica et Cosmochimica Acta* **57**, 277-283 (1993).
2. U. Mann, D. J. Frost, D. C. Rubie, H. Becker, A. Audétat, Partitioning of Ru, Rh, Pd, Re, Ir and Pt between liquid metal and silicate at high pressures and high temperatures-Implications for the origin of highly siderophile element concentrations in the Earth's mantle. *Geochimica et Cosmochimica Acta* **84**, 593-613 (2012).
3. E. Médard, M. W. Schmidt, M. Wälle, N. S. Keller, D. Günther, Platinum partitioning between metal and silicate melts: Core formation, late veneer and the nanonuggets issue. *Geochimica et Cosmochimica Acta* **162**, 183-201 (2015).
4. S. D. Sourcebook. (Gordon and Breach Science Publishers, New York, 1988).
5. N. R. Bennett, J. M. Brenan, K. T. Koga, The solubility of platinum in silicate melt under reducing conditions: Results from experiments without metal inclusions. *Geochimica et Cosmochimica Acta* **133**, 422-442 (2014).
6. W. Ertel, M. J. Walter, M. J. Drake, P. J. Sylvester, Experimental study of platinum solubility in silicate melt to 14 GPa and 2273 K: Implications for accretion and core formation in Earth. *Geochimica et Cosmochimica Acta* **70**, 2591-2602 (2006).
7. A. Borisov, H. Palme, Solubilities of noble metals in Fe-containing silicate melts as derived from experiments in Fe-free systems. *American Mineralogist* **85**, 1665-1673 (2000).
8. P. J. Sylvester, S. M. Eggins, Analysis of Re, Au, Pd, Pt and Rh in NIST glass certified reference materials and natural basalt glasses by laser ablation ICP-MS. *Geostandards Newsletter* **21**, 215-229 (1997).
